# Outcomes of maze procedure and mitral valve surgery in atrial functional mitral regurgitation: a retrospective study

**DOI:** 10.1186/s13019-024-02858-w

**Published:** 2024-07-10

**Authors:** Kyungsub Song, Woo Sung Jang, Yun Seok Kim, Jonghoon Yoo

**Affiliations:** 1 Department of Thoracic and Cardiovascular Surgery, Keimyung University Dongsan Medical Center, Keimyung University College of Medicine, 1035, Dalgubeol-daero, Dalseo-gu, Daegu, 42601 Republic of Korea; 2 Department of Emergency Medicine, Keimyung University Dongsan Medical Center, Keimyung University College of Medicine, 1035, Dalgubeol-daero, Dalseo-gu, Daegu, 42601 Republic of Korea

**Keywords:** Atrial fibrillation, Mitral valve insufficiency, Maze procedure

## Abstract

**Background:**

Atrial functional mitral regurgitation (AFMR) is a newly discovered condition associated with longstanding atrial fibrillation. This retrospective study aimed to analyze the outcomes of the maze procedure and mitral regurgitation (MR) surgery in AFMR and atrial fibrillation in comparison with those in degenerative MR (DMR).

**Methods:**

Patients who underwent mitral valve repair/replacement with a maze procedure at a hospital (July 2012–August 2021) were included. We excluded patients aged below 18 years undergoing concomitant coronary artery bypass grafting or atrial septal defect repair and those with MR etiology other than ARMR or DMR.

**Results:**

We included 35 patients with AFMR and 50 patients with DMR. Patient characteristics and postoperative outcomes were not significantly different between the two groups. Long-term outcomes revealed no significant differences in the ratio of cardiac mortality, stroke, or hospital readmission. However, after the maze procedure, the sinus rhythm restoration rate was significantly lower (62% vs. 28.5%, *p* < 0.001), a junctional rhythm state (*p* < 0.001) and permanent pacemaker insertion for sick sinus syndrome (SSS) (*p* = 0.03) were significantly more common in AFMR than DMR. On postoperative transthoracic echocardiography (TTE), the pulmonary artery systolic pressure was significantly less decreased in the AFMR group than in the DMR group compared with that on preoperative TTE (*p* = 0.04).

**Conclusions:**

AFMR showed excellent mitral valve surgery outcomes, similar to DMR, but had a significantly higher risk of pacemaker insertion for SSS after the maze procedure.

**Supplementary Information:**

The online version contains supplementary material available at 10.1186/s13019-024-02858-w.

## Background

Atrial functional mitral regurgitation (AFMR) is a newly identified condition that is usually combined with longstanding atrial fibrillation (AF), especially after an AF duration of over ten years [1, 2]. In AFMR, the left atrium (LA) and mitral annulus are dilated without left ventricular (LV) dysfunction as seen in AF [3–5]. Therefore, the surgical results of mitral regurgitation (MR) are better in AFMR than in ventricular functional MR (VFMR), which occurs secondary to LV dysfunction and dilatation. However, the surgical outcomes in AFMR still need to be more understood, given that surgical and hemodynamic change results of AFMR are uncommon [6, 7]. Rhythm control in AFMR is especially important in improving patients’ prognoses [8] but reports about maze procedure results are rare in AFMR.

In this study, we analyzed the outcomes of the maze procedure and MR surgery of AFMR by comparing them with those of degenerative MR (DMR), which is the most common primary MR and has favorable outcomes [9, 10].

## Methods

### Patient population

This study included patients with MR who underwent cardiac surgery, including mitral valve (MV) repair/replacement with a maze procedure at a single medical center from July 2012 to August 2021. We excluded patients aged below 18 years and diagnosed with either of the following MR etiologies: rheumatic heart disease, infective endocarditis, VFMR, systolic anterior motion in hypertrophic cardiomyopathy, and Barlow’s disease (Supplemental Fig. 1). We also excluded patients who underwent concomitant coronary artery bypass grafting surgery or atrial septal defect repair with MR surgery.

### Criteria of AFMR and DMR

The definition of AFMR was based on the criteria presented in recent related journals [11]. All patients were divided into AFMR and DMR groups according to their preoperative transthoracic echocardiography (TTE) and operative records. The criteria of AFMR were as follows: normal LV size, geometry (LV volume ≤ 85 mL/m^2^ [male] or ≤ 78 mL/m^2^ [female]), and shape; preserved regional and global function (LV ejection fraction ≥ 50%); dilated LA size (LA volume index ≥ 40 mL/m^2^ and LA diameter ≥ 40 mm on the maximal anteroposterior diameter of the LA measured with M-mode at ventricular end-systole); annular dilation and flattening with normal leaflet motion (Carpentier type I MR). Conversely, Carpentier type II MR (billowing, flail leaflets, or chordae rupture without rheumatic heart disease or endocarditis) indicated DMR [12, 13].

### Operative techniques and maze procedure

We first attempted mitral valve repair rather than mitral valve replacement in cardiac surgery for MR unless there was severe LV dilatation or leaflet thickness. According to the operator’s preference, we used a semirigid-complete ring in mitral valve repair. We perform mitral valve replacement if the result of mitral valve repair is unsatisfactory. A partial ring was used to repair the tricuspid valve following our indications for TAP, more-than-moderate tricuspid regurgitation (TR), or annular dilation (diameter of > 4.0 cm) based on preoperative echocardiography.

A modified Cox maze procedure with cryoablation was performed with antegrade cardioplegia in the maze procedure, according to the principles described in a previous study [14]. Criteria for patient selection for the maze procedure included established predictors for regaining normal heart rhythm, as reported in previous studies [15, 16], which involved evaluating the size and fibrosis of the left atrium, the history of AF, and any additional surgeries. The pattern of left atrial lesions involved three specific ablation lines, each extending from the top and bottom of the left atriotomy toward the left atrial appendage for isolating the pulmonary veins and the endocardium mitral line from the left atriotomy to the posterior mitral annulus. The approach for the right atrium included three lines: running from the superior to the inferior vena cava, the T lesion from the intercaval lesion extending to the TV annulus, and the right atrial appendage lesion from the right atriotomy extending to the TV annulus. The left atrial appendage was closed in every patient in this study through internal obliteration. The specific configurations of lesions were generally consistent throughout the study period despite minor adjustments.

### Echocardiography and rhythm follow-up

All patient characteristics and outcome data were extracted from our medical center’s electronic medical records. Preoperative TTE was defined as the latest results of TTE before the surgery. We routinely performed TTE before hospital discharge and one year postoperatively; each was defined as postoperative TTE and 1-year follow-up TTE, respectively. Early mortality was defined as mortality within 30 days after surgery.

During the follow-up period, ECG was performed in the outpatient clinic at 1, 3, and 6 months postoperatively and then every six months. No AF recurrence in at least two consecutive ECGs indicated sinus rhythm restoration. All other rhythms, including AF, atrial flutter, ectopic atrial arrhythmia, junctional rhythm, and cardiac rhythm of permanent pacemaker without atrioventricular synchrony, defined failure of sinus rhythm restoration.

### Outcomes

The primary outcome was freedom from major adverse cardiac events (MACE), including cerebral infarction, readmission for heart failure, and cardiac mortality. Secondary outcomes were the maze procedure results (ratio of AF absence and sinus rhythm maintenance), ratio of permanent pacemaker insertion, and postoperative results, including early mortality, cardiac hemodynamic changes, and MR recurrence rate.

### Statistical analysis

Normally distributed variables were compared using independent t-tests and presented as means. In contrast, categorical variables were compared using Pearson’s chi-square or Fisher’s exact test and presented as numbers (percentages). All statistical tests were 2-sided, with an alpha level of 0.05.

The ratio of freedom from MACE, all-cause mortality, and MR recurrence rate were estimated using the Kaplan–Meier method with the log-rank test. The repeated-measures binary outcome of the maze procedure was analyzed using a generalized estimation equation model, and changes in cardiac hemodynamics were evaluated using a linear mixed model. Predictors of the maze procedure results (ratio of freedom from AF and maintenance of sinus rhythm at discharge from the hospital) were identified through logistic regression. Variables with *p* < 0.2 in the univariate analysis were included in the multivariate analysis [17], and models in the multivariate analysis were selected using backward elimination. The results are reported as odds ratio (OR) in the logistic regression test and as hazard ratio (HR) in the Cox regression test with a 95% confidence interval (CI). Statistical data were analyzed using SPSS software version 29.0 (IBM-SPSS Inc., Armonk, NY, USA) and R statistical software version 4.0.2 (R Foundation for Statistical Computing, Vienna, Austria).

## Results

### Patient characteristics

We included 35 patients with AFMR (mean age, 68.0 years) and 50 patients with DMR (mean age, 64.4 years) (Table 1). The DMR group had significantly more males than the ARMR group (42% vs. 40%, *p* = 0.05). Concomitant TAP was performed in 65.7% (23/35) and 70% (35/50) of the AFMR and DMR groups, respectively (*p* = 0.68). Aortic valve replacement was also performed in 2 patients with AFMR for moderate aortic stenosis (AS) and in 4 patients with DMR for moderate AS (3 patients) and severe AS (1 patient). In addition, two patients with DMR underwent concomitant aortic valve plasty (aortic valve resuspension) for moderate aortic regurgitation.

**Table 1:** Patient characteristics

	AFMR (*n* = 35)	DMR (*n* = 50)	*p* value
Sex, male, n (%)	14 (40)	31 (62)	***0.05***
Age, year	68.0 ± 8.1	64.4 ± 0.5	0.09
Body surface area, kg/m^2^	1.69 ± 0.19	1.67 ± 0.26	0.79
Left atrial appendage obliteration, n (%)	13 (37.1)	15 (30)	0.49
Concomitant operations, n (%)			
Tricuspid annuloplasty	23 (65.7)	35 (70)	0.68
Aortic valve replacement	2 (5.7)	4 (8)	> 0.99
Aortic valve plasty	0	2 (4)	0.17
Atrial fibrillation type, n (%)			
Paroxysmal	1 (2.9)	4 (8)	0.64
Permanent	34 (97.1)	46 (92)	
Fine fibrillatory wave (< 1 mm)	23 (65.7)	24 (48)	0.11
Comorbidity, n (%)			
Heart failure	15 (42.9)	13 (26)	0.10
Coronary artery disease	5 (14.3)	4 (8)	0.48
Cerebral infarction	4 (11.4)	7 (14)	> 0.99
Diabetes mellitus on medication	3 (8.6)	11 (22)	0.14
Hypertension on medication	17 (48.6)	27 (54)	0.62
Chronic obstructive lung disease	0	1 (2)	> 0.99
Chronic kidney disease	2 (5.7)	5 (10)	0.70
Values on preoperative TTE			
Ejection fraction, %	56.1 ± 10.5	58.6 ± 10.3	0.29
Left ventricular end-diastolic diameter, cm	5.5 ± 0.6	5.7 ± 0.9	0.14
Left atrial diameter, cm	6.1 ± 0.8	5.7 ± 0.9	***0.03***
Left ventricular volume index, mL/m^2^	63.7 ± 17.1	77.3 ± 30.6	***0.02***
Left atrial volume index, mL/m^2^	132.9 ± 52.1	120.6 ± 59.3	0.34
Tricuspid valve annulus size, mm	4.1 ± 0.5	4.0 ± 0.6	0.43
Pulmonary artery systolic pressure, mmHg	41.7 ± 11.3	44.5 ± 16.5	0.37

Significant p values are shown in italics and bold

TTE = transthoracic echocardiography

In preoperative TTE, the mean LA diameter was significantly larger in the AFMR group than in the DMR group (6.1 ± 0.8 cm vs. 5.7 ± 0.9 cm, *p* = 0.03). However, the LV volume index was significantly larger in the DMR group than in the AFMR group (77.3 ± 30.6 mL/m^2^ vs. 63.7 ± 17.1 mL/m^2^, *p* = 0.02) (Table 1).

### Operative data

MV repair was performed in 88.6% (31/35) of the AFMR group and 84% (42/50) of the group (*p* = 0.75) (Table 2). Early mortality occurred in 2 patients with AFMR (after MV repair with TAP and after MV repair alone) and three patients with DMR (all after MV replacement). Other short-term postoperative outcomes were not significantly different between the two groups. Regarding postoperative TTE results, the mean LA diameter remained significantly larger in the AFMR group than in the DMR group (5.42 ± 0.86 cm vs. 4.98 ± 0.70 cm, *p* = 0.01). Other postoperative TTE results showed no significant difference between the two groups.

**Table 2:** Results of the surgery

	AFMR (*n* = 35)	DMR (*n* = 50)	*p* value
Mitral valve replacement, n (%)	4 (11.4)	8 (16)	0.75
Mitral annuloplasty, n (%)	31 (88.6)	42 (84)	
Mechanical valve implantation (%)	1 (2.9)	3 (6)	0.64
Aortic cross clamp time, min	125.9 ± 41.0	125.9 ± 55.3	> 0.99
Cardiopulmonary bypass time, min	166.9 ± 49.8	165.7 ± 74.8	0.94
Bleeding control, n (%)	1 (2.9)	3 (6)	0.64
Early mortality, n (%), within 30 days	2 (5.7)	3 (6)	> 0.99
Hospital stay, days	15.6 ± 7.6	16.2 ± 10.7	0.79
Follow-up period, months	46.5 ± 29.3	51.1 ± 30.4	0.49
Values on postoperative TTE			
Ejection fraction, %	55.1 ± 10.3	55.6 ± 11.7	0.82
Left ventricular end-diastolic diameter, cm	5.08 ± 0.55	5.25 ± 0.55	0.17
Left atrial diameter, cm	5.42 ± 0.86	4.98 ± 0.70	***0.01***
Left ventricular volume index, mL/m^2^	55.5 ± 12.1	61.1 ± 18.2	0.12
Left atrial volume index, mL/m^2^	97.1 ± 28.5	90.2 ± 34.9	0.37

Significant p values are shown in italics and bold

AFMR = atrial functional mitral regurgitation; DMR = degenerative mitral regurgitation

### Outcomes

**Fig. 1:**
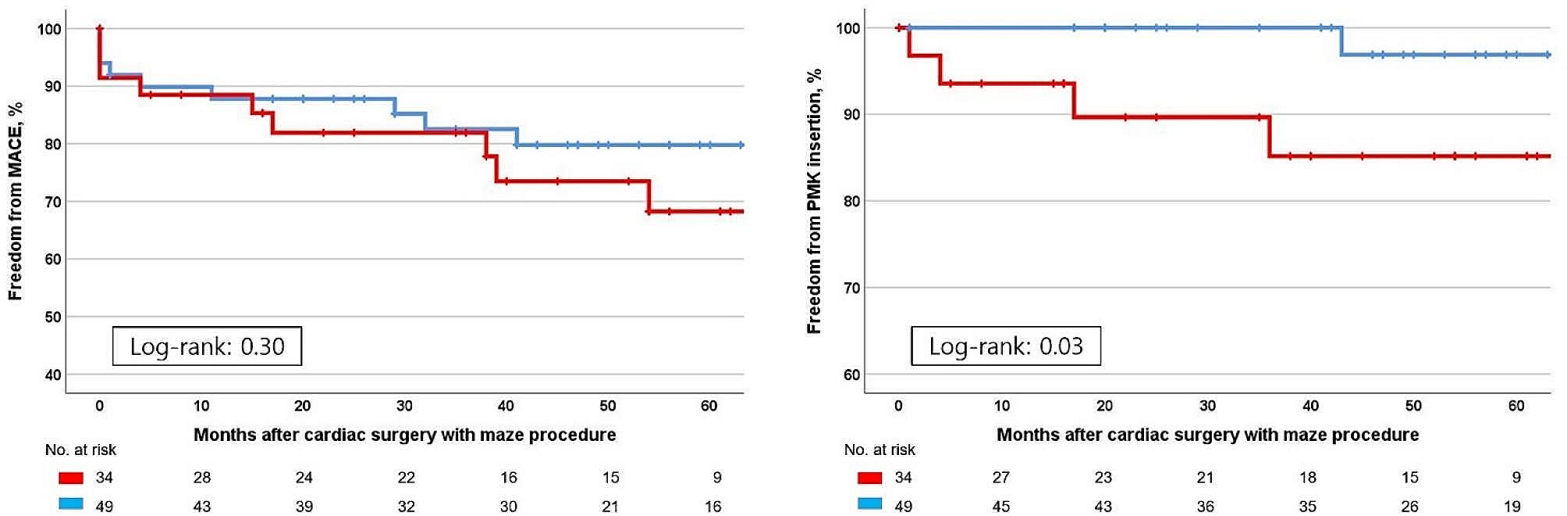
Results of major adverse cardiac events and ratio of permanent pacemaker insertion

The ratio of freedom from MACE at 1, 3, and 5 years postoperatively was 88.5%, 81.9%, and 68.2% in the AFMR group and 87.8%, 82.5%, and 79.8% in the DMR group, respectively (*p* = 0.30) (Fig. 1). The postoperative results of freedom from stroke (*p* = 0.07), readmission for heart failure (*p* = 0.14), and cardiac mortality (*p* = 0.19) were not significantly different between the two groups. However, significantly more patients with AFMR needed permanent pacemaker insertion than those with DMR (*p* = 0.03).

**Fig. 2:**
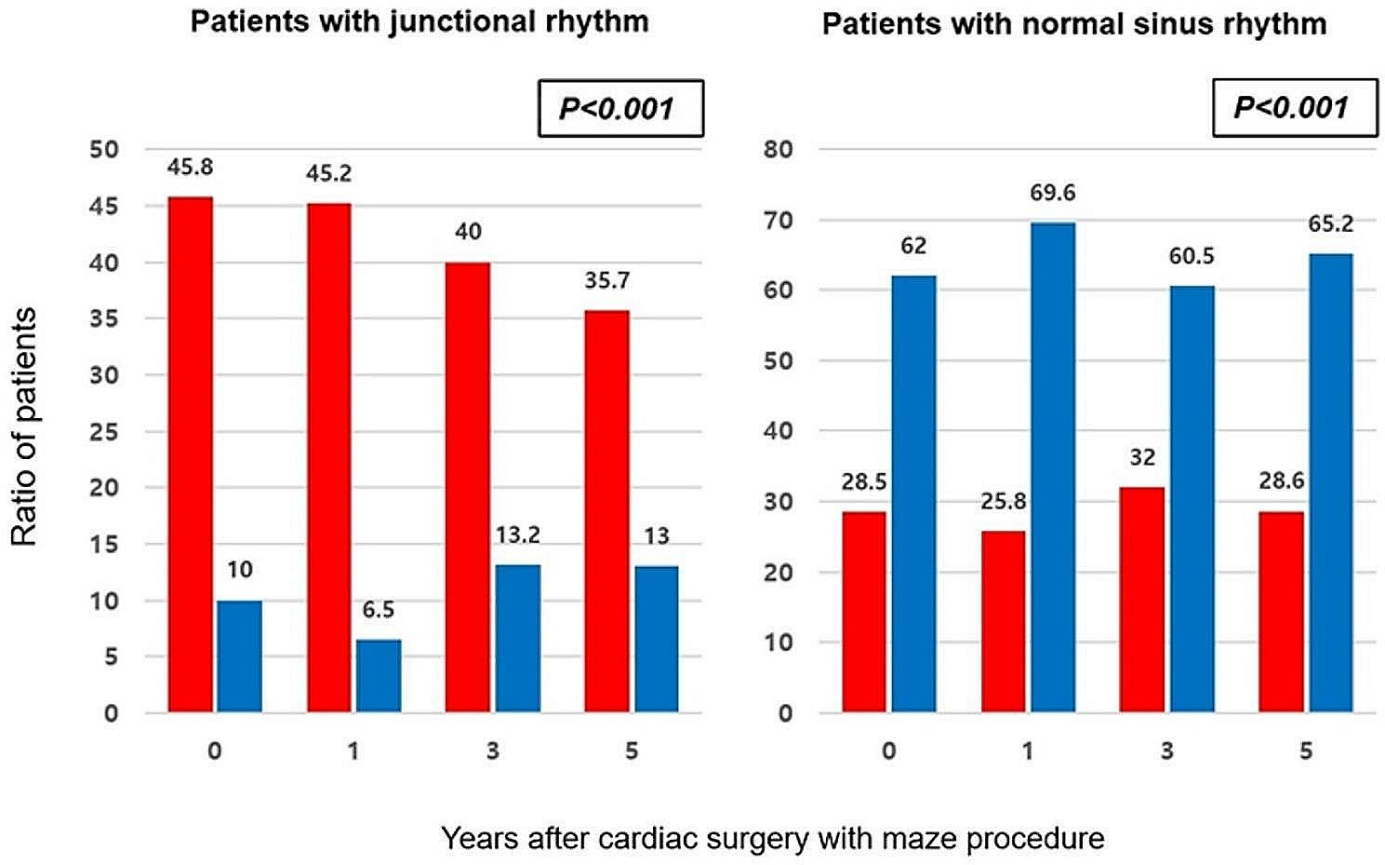
Results of the maze procedure

Figure 2 shows the maze procedure results. In AFMR, the ratio of sinus rhythm restoration was significantly lower (*p* = 0.001), and a junctional rhythm was significantly more common than in those with DMR (*p* = 0.001) during the follow-up period. Multivariate analysis revealed that a fine fibrillatory P wave in preoperative ECG was a significant risk factor for AF recurrence after the maze procedure (OR: 0.289, 95% CI: 0.089–0.943, *p* = 0.04) (Supplementary Table 1). Furthermore, AFMR (OR: 2.907, 95% CI: 1.107–7.634, *p* = 0.03) and fine fibrillatory P wave (OR: 2.849, 95% CI: 1.112–7.299, *p* = 0.04) were significant risk factors for junctional rhythm occurring after the maze procedure (Supplementary Table 2).

In the latest follow-up data, the percentage of patients with anticoagulation therapy in the DMR and AFMR groups was 48.7% and 88.5% (*p* = 0.001), respectively, while using antiarrhythmic drugs (AAD) was 20.5% and 7.7% (*p* = 0.293), respectively. Despite the lower sinus rhythm restoration rate in the AFMR group, the reason for the fewer patients using AAD is that many patients in the AFMR group are in a state of bradycardia or permanent AF.

### Postoperative hemodynamic changes

**Fig. 3:**
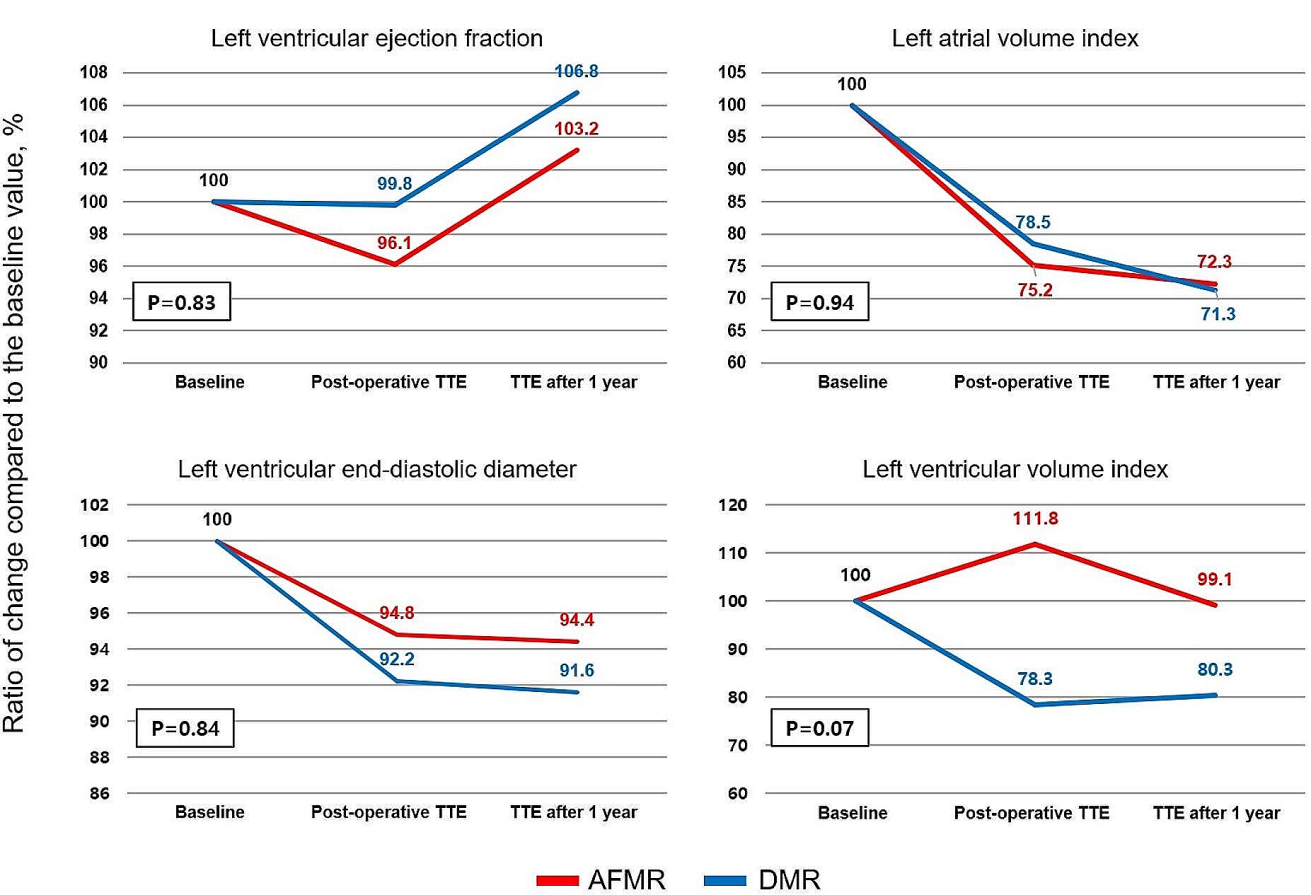
Hemodynamic changes of the left ventricle and atrium after surgery

**Fig. 4:**
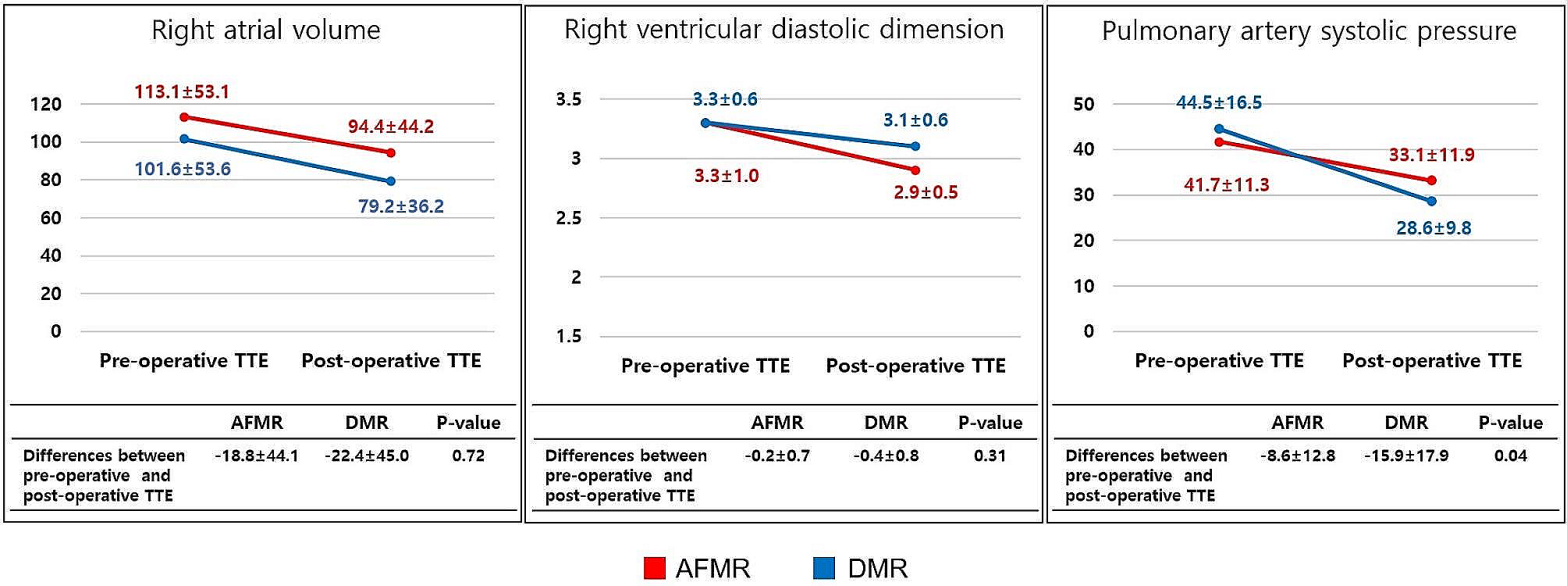
Hemodynamic changes of the right ventricle and atrium after surgery

After surgery with a maze procedure for MR, all hemodynamic values of the left heart improved in both groups, but their differences were not significant (Fig. 3). Regarding the changes in right heart hemodynamics, both groups showed improvement in right atrial volume, right ventricular diastolic dimension, and pulmonary artery systolic pressure (PASP) after surgery (Fig. 4). The PASP was significantly more decreased in DMR than in AFMR (− 15.9 ± 17.9 vs. −8.6 ± 12.8, *p* = 0.04).

### Long-term results of mitral annuloplasty

**Fig. 5:**
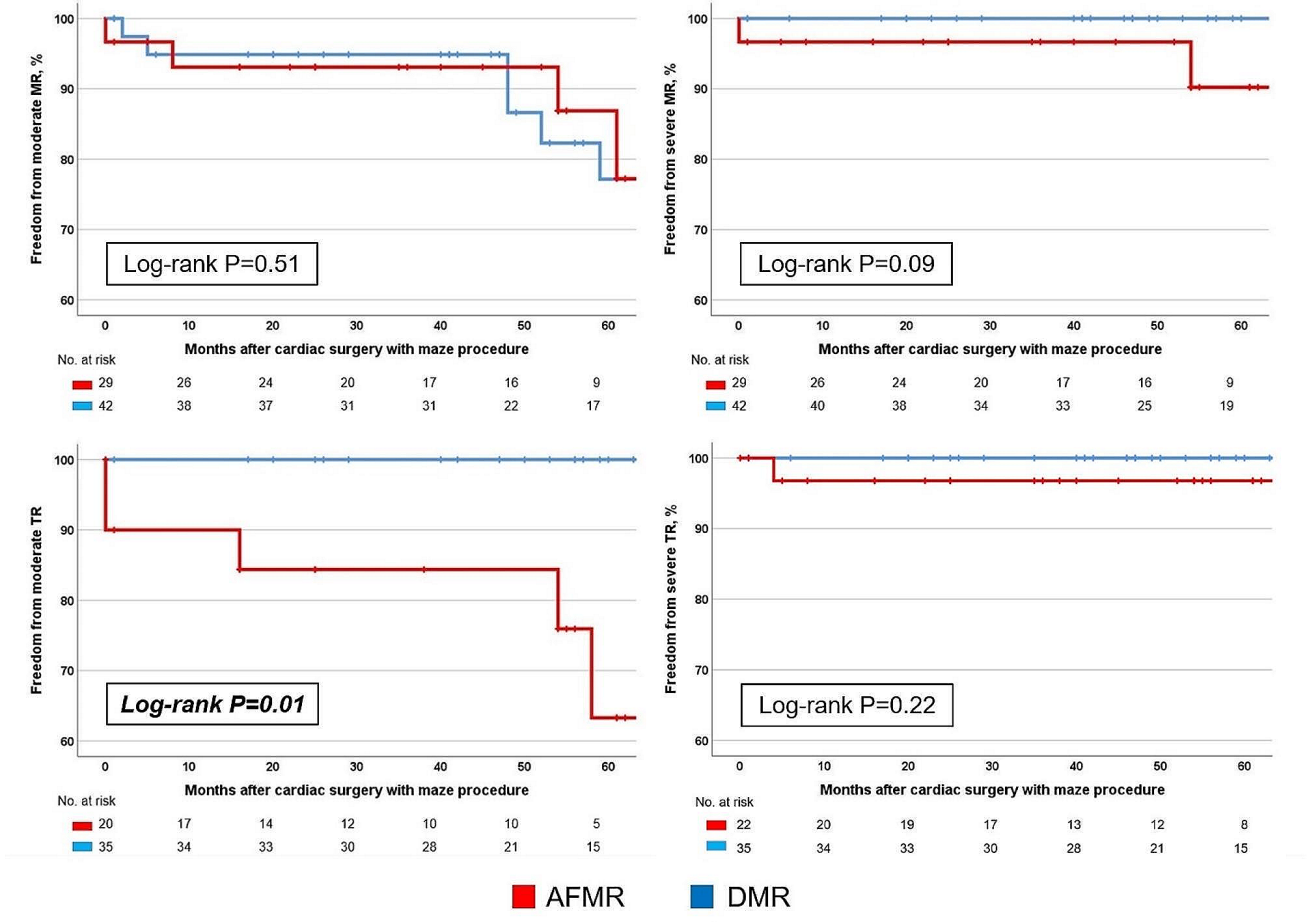
Ratio of mitral regurgitation (MR) and tricuspid regurgitation (TR) recurrence after surgery

The ratio of freedom from MR recurrence (more-than-moderate MR) after MV repair was not significantly different between 2 groups (*p* = 0.51) (Fig. 5). During the follow-up period, severe MR recurred in 2 patients with AFMR (1 week and 54 months postoperatively in each case) and one patient with DMR (109 months postoperatively). The patient in whom severe MR recurred one week postoperatively underwent redo–MV replacement one month after the first surgery.

In the total cohort, including all patients who underwent MV surgery with or without TAP, the ratio of freedom from TR (more-than-moderate) after surgery was significantly higher in the AFMR than in the DMR group (*p* = 0.02) (Supplemental Fig. 2).

## Discussion

This report has two key findings. First, significantly more patients with AFMR showed a junctional rhythm after the maze procedure than those with DMR; this finding was related to a higher incidence of permanent pacemaker insertion during the follow-up period. Second, the short- and mid-term outcomes of AFMR were favorable.

In this article, significantly more patients with AFMR showed a junctional rhythm and lower sinus rhythm restoration rate after the maze procedure. AFMR occurs in 2.4–66.7% of patients with AF [1, 8], and the prevalence of AFMR increases proportionately with the longstanding AF [1]. Although Wagner et al. [15] reported that 61% of patients with AFMR sustained normal sinus rhythm at their latest follow-up after the maze procedure, patients with AFMR have a high risk of developing SSS because longstanding AF has an effect of diffusing atrial remodeling with the extensive loss of automatic pacemaker tissue and widespread impairment of sinoatrial conduction [19, 20]. Hence, patients with AFMR have a high risk for longstanding AF with sinoatrial conduction impairment and an extensive loss of automatic pacemaker tissue; this phenomenon leads to a junctional rhythm after the maze procedure. It explains why more patients with AFMR needed permanent pacemaker insertion than those with DMR.

In the current study, the outcome of MV repair in AFMR was as good as that in DMR. The study of Wagner et al. [21] currently has the largest number of patients reporting the surgical results of AFMR. In their report, the freedom from MR was more than moderate and was nearly 95% at their latest follow-up, and the reintervention rate of AFMR after MV repair was less than 2%. In the present study, only 1 out of 30 (3.3%) patients with AFMR underwent reintervention after MV repair. The mean long-term survival rate of patients with AFMR was good and better than that of patients with VFMR [18, 22], and mitral repair is a good treatment option for AFMR [18, 21].

However, the PASP was significantly less improved in the AFMR group than in the DMR group, and it caused a higher occurrence rate of TR after operation. The reason could be the poor LA function of AFMR from a larger LA volume and the fewer number of patients with sinus rhythm (more patients with nonfunctional LA). Consequently, the afterload of the right ventricle (PASP) was significantly higher in the AFMR group than in the DMR group. Previous studies [18, 23] also showed a high incidence of right ventricular dysfunction in AFMR.

This study has several limitations. First, our data were retrospectively analyzed in a small number of patients, and the analysis lacks several significant factors, such as AF duration, that could influence the outcomes. Although several results showed large differences in ratios, no significant differences were observed because of the limited number of patients. This study is not a randomized controlled trial; thus, it has limited statistical power. Second, we did not have long-term echocardiographic follow-up data for all patients. Thus, our study results do not accurately reflect the long-term operative results of AFMR. Third, follow-up screening for AF recurrence was based on serial 12-lead ECG rather than 24-hour Holter monitoring. Hence, our results were possibly overestimated because asymptomatic paroxysmal AF may have been present during the follow-up period.

## Conclusions

MV surgical outcomes for AFMR were excellent and comparable to those of DMR. However, in AFMR, the maze procedure’s result was inferior to that of DMR, and more patients needed permanent pacemaker insertion postoperatively; this result was related to a higher ratio of patients with a junctional rhythm. As a result, the left heart function and mortality outcomes were favorable in AFMR, but the maze procedure result was not good. Additional prospective studies with more comprehensive follow-ups are needed.

### Electronic supplementary material

Below is the link to the electronic supplementary material.

Supplementary Material 1

Supplementary Material 2

Supplementary Material 3

Supplementary Material 4

## Data Availability

No datasets were generated or analysed during the current study.

## Abbreviations

AFMR: atrial functional mitral regurgitation
AF: atrial fibrillation
LA: left atrium
LV: left ventricle
MR: mitral regurgitation
VFMR: ventricular functional mitral regurgitation
DMR: degenerative mitral regurgitation
MV: mitral valve
TTE: transthoracic echocardiography
TAP: tricuspid annuloplasty
TR: tricuspid regurgitation
MACE: major adverse cardiac event
OR: odds ratio
HR: hazard ratio
CI: confidence interval
