# Supplementary figures and images for: Outcomes of maze procedure and mitral valve surgery in atrial functional mitral regurgitation: a retrospective study
Source: J Cardiothorac Surg. 2024 Jul 10;19:433. doi: 10.1186/s13019-024-02858-w (PMC11234554; doi:10.1186/s13019-024-02858-w)

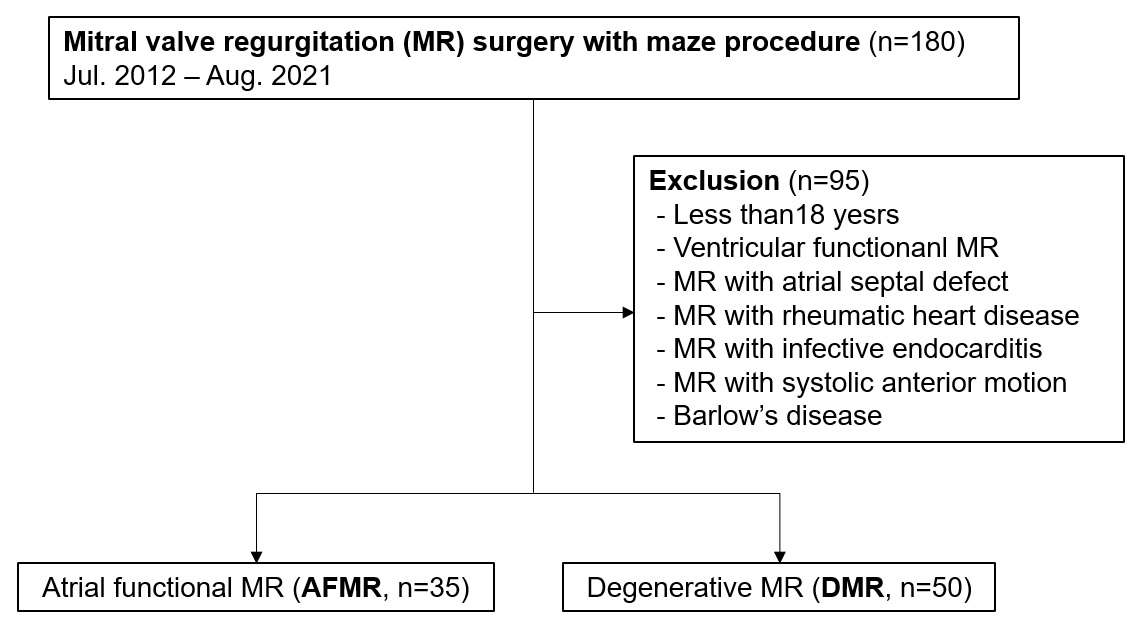

Supplement: Supplementary file 1 — Supplementary Material 1 [file 13019_2024_2858_MOESM1_ESM.jpg]

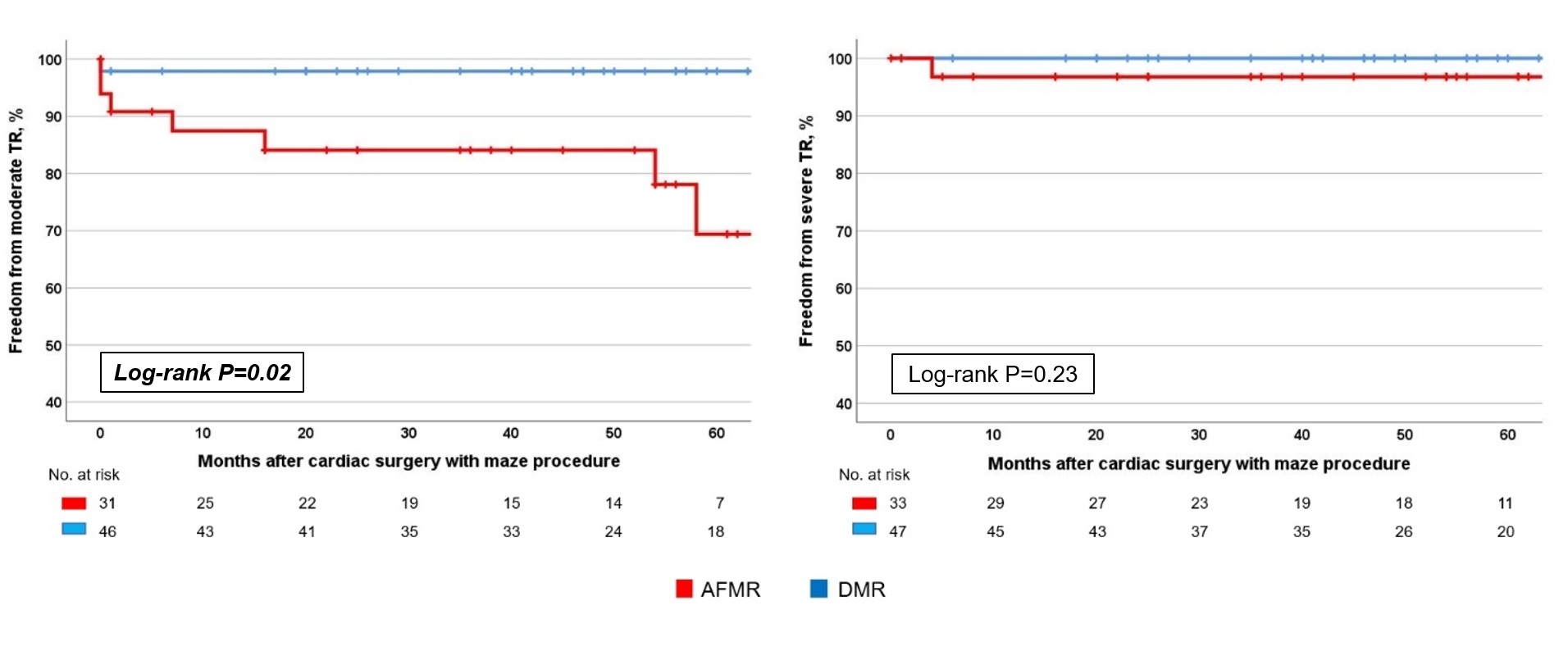

Supplement: Supplementary file 2 — Supplementary Material 2 [file 13019_2024_2858_MOESM2_ESM.jpg]
