# Supplementary material for: Outcomes of maze procedure and mitral valve surgery in atrial functional mitral regurgitation: a retrospective study
Source: J Cardiothorac Surg. 2024 Jul 10;19:433. doi: 10.1186/s13019-024-02858-w (PMC11234554; doi:10.1186/s13019-024-02858-w)
Supplement: Supplementary file 3 — Supplementary Material 3 [file 13019_2024_2858_MOESM3_ESM.docx]

Supplementary Table 1. Logistic regression analysis of factors for freedom from postoperative atrial fibrillation on electrocardiography (At the first visit to the outpatient clinic after discharge from the hospital).

|  | Univariate | |  | Multivariate | | |
| --- | --- | --- | --- | --- | --- | --- |
|  | OR (95% CI) | *P* | OR (95% CI) | | *P* |  |
| Sex, male | 0.464 (0.175–1.235) | 0.12 | 1.931 (0.660–5.646) | | 0.23 |  |
| Age | 1.016 (0.965–1.070) | 0.54 |  | |  |  |
| AFMR | 0.890 (0.335–2.364) | 0.82 |  | |  |  |
| LAA obliteration | 0.854 (0.304–2.398) | 0.77 |  | |  |  |
| Fine fibrillatory wave | 4.098 (1.351–12.346) | 0.01 | 0.289 (0.089–0.943) | | ***0.04*** |  |
| Chronic kidney disease | 8.333 (1.488–47.619) | 0.02 | 0.255 (0.038–1.706) | | 0.16 |  |
| Ejection fraction | 0.975 (0.929–1.024) | 0.31 |  | |  |  |
| LVEDD | 1.678 (0.863–3.333) | 0.13 | 0.720 (0.337–1.549) | | 0.40 |  |
| LAD | 1.145 (0.653–2.008) | 0.64 |  | |  |  |

Significant p values are shown in italics and bold.

AFMR = atrial functional mitral regurgitation; CI = confidence interval; LAA = left atrial appendage; LAD = left atrial diameter; LVEDD = left ventricular end-diastolic diameter; OR = odds ratio.
