# Supplementary material for: Outcomes of maze procedure and mitral valve surgery in atrial functional mitral regurgitation: a retrospective study
Source: J Cardiothorac Surg. 2024 Jul 10;19:433. doi: 10.1186/s13019-024-02858-w (PMC11234554; doi:10.1186/s13019-024-02858-w)
Supplement: Supplementary file 4 — Supplementary Material 4 [file 13019_2024_2858_MOESM4_ESM.docx]

Supplementary Table 2. Logistic regression analysis of factors for postoperative junctional rhythm on electrocardiography (At the first visit to the outpatient clinic after discharge from the hospital).

|  | Univariate | | | Multivariate | |
| --- | --- | --- | --- | --- | --- |
|  | OR (95% CI) | *P* | | OR (95% CI) | *P* |
| Sex, male | 0.792 (0.337–1.859) | 0.59 |  | |  |
| Age | 1.036 (0.990–1.085) | 0.22 | |  |  |
| AFMR | 3.559 (1.427–8.850) | 0.006 | | 2.907 (1.107–7.634) | ***0.03*** |
| LAA obliteration | 0.629 (0.253–1.565) | 0.32 | |  |  |
| Fine fibrillatory wave | 3.390 (1.385–8.333) | 0.008 | | 2.849 (1.112–7.299) | ***0.03*** |
| Chronic kidney disease | 2.770 (0.507–15.152) | 0.24 | |  |  |
| Ejection fraction | 1.028 (0.985–1.072) | 0.21 | |  |  |
| LVEDD | 0.778 (0.445–1.362) | 0.38 | |  |  |
| LAD | 1.650 (0.974–2.801) | 0.06 | | 1.302 (0.740–2.294) | 0.36 |

Significant p values are shown in italics and bold.

AFMR = atrial functional mitral regurgitation; CI = confidence interval; LAA = left atrial appendage; LAD = left atrial diameter; LVEDD = left ventricular end-diastolic diameter; OR = odds ratio.
